# Non-targeted and targeted analysis of collagen hydrolysates during the course of digestion and absorption

**DOI:** 10.1007/s00216-019-02323-x

**Published:** 2019-12-24

**Authors:** Anne J. Kleinnijenhuis, Frédérique L. van Holthoon, Annet J.H. Maathuis, Barbara Vanhoecke, Janne Prawitt, Fabien Wauquier, Yohann Wittrant

**Affiliations:** 1 Triskelion, Utrechtseweg 48, 3704 HE Zeist, the Netherlands; 2 Rousselot BVBA, Meulestedekaai 81, 9000 Ghent, Belgium; 3 INRA, Université Clermont Auvergne, UMR 1019 de Nutrition Humaine, CRNH Auvergne, 63000 Clermont-Ferrand, France

**Keywords:** LC-MS, Hydrolysate, Bioaccessibility, Bioavailability, Collagen, Absorption

## Abstract

Protein hydrolysates are an important part of the human diet. Often, they are prepared from milk, soy, or collagen. In the present study, four different collagen hydrolysates were tested, varying in the average molecular weight and the animal source. Three types of samples, the dissolved start products, in vitro generated dialysates (containing the digested components that are potentially available for small intestinal absorption), and human serum collected after product ingestion, were analyzed using LC-MS to compare the state of the hydrolysates before and after absorption, i.e., uptake into the blood. It was found that the composition of the collagen hydrolysates prior to and after ingestion was highly complex and dynamic, which made it challenging to predefine a strategy for a targeted analysis. Therefore, we implemented a new analytical approach to first map hydrolysate data sets by performing non-targeted LC-MS analysis followed by non-targeted and targeted data analysis. It was shown that the insight gained by following such a top down (data) analytical workflow could be crucial for defining a suitable targeted setup and considering data trends beyond the defined targets. After having defined and performed a limited targeted analysis, it was found that, in our experimental setup, Hyp-Gly and especially Pro-Hyp contributed significantly as carrier to the total Hyp increase in blood after ingestion of collagen hydrolysate.

**Electronic supplementary material:**

The online version of this article (10.1007/s00216-019-02323-x) contains supplementary material, which is available to authorized users.

**Figure:**
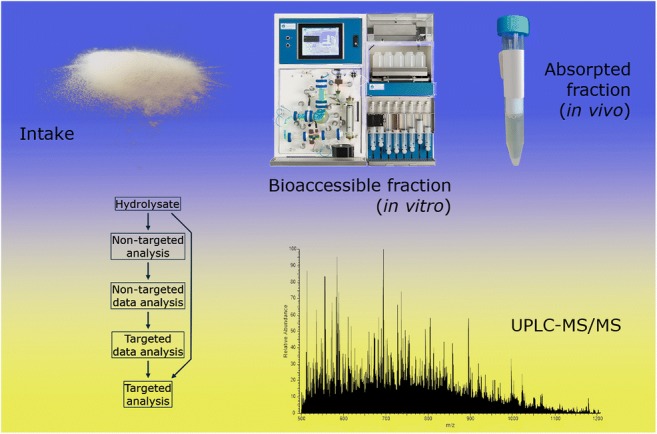
Graphical abstract

## Introduction

Protein hydrolysates are an important part of the human diet. The protein sources for hydrolysates used in nutrition are frequently obtained from milk (e.g., in infant formula), or from soy, which are basic ingredients in many food applications. For subjects suffering from reduced absorptive capacity, it can be an option to consume protein in hydrolyzed form to facilitate the uptake of essential amino acids [1]. Hydrolysis can also disrupt protein epitopes involved in particular food allergies [1]. Compared with intact protein, the ingestion of protein hydrolysate accelerates protein digestion and absorption in the gut, increases amino acid availability after passage through the stomach, and tends to increase the incorporation rate of dietary amino acids into skeletal muscle protein [2]. Collagen is another frequently used protein source for hydrolysates. It has been reported that dietary supplementation with collagen hydrolysates can support joint, bone [3, 4], and skin health [5, 6]. These and other putative health benefits are an active field of research.

Collagen hydrolysate is prepared from collagen or gelatin by chemical or enzymatic hydrolysis under controlled conditions [7]. Collagen extraction from tissues such as bone or skin is usually performed under acidic or alkaline conditions and/or heating, which will result in protein denaturation and in non-specific cleavage of peptide bonds. The enzymes used for the production of protein hydrolysates such as collagen hydrolysate often have a broad specificity [8–10]. Therefore, after these typical treatments to obtain collagen hydrolysate, peptides with varying lengths and varying C- and N-terminal amino acids are formed. Hydrolysate peptides, when ingested, will be exposed to acidic conditions in the stomach and to pepsin, an enzyme with broad specificity [11]. After passage to the duodenum, the hydrolysate peptides will further be exposed to trypsin and chymotrypsin secreted by the pancreas. Trypsin cleaves C-terminally of the basic residues lysine and arginine; chymotrypsin cleaves C-terminally of phenylalanine, tyrosine, and tryptophan [12, 13]. Finally, at the brush border, peptidases are present that cleave peptides down to small peptides and amino acids, ready for uptake into enterocytes and transport to the blood. The end product of protein digestion is a complex mixture of primarily dipeptides and tripeptides, along with individual amino acids [14].

The analysis of protein hydrolysates using ultra-performance liquid chromatography-mass spectrometry (UPLC-MS) can be complex. In this section, several underlying considerations are summarized and they are discussed in more detail in the Electronic Supplementary Material (ESM). The number of different molecular species in protein hydrolysates (observed with MS) can be high due to (1) the production of hydrolysates by chemical hydrolysis and/or enzymes with broad specificity, (2) the presence of partial modification sites, (3) the (original) presence of non-linear structure elements, and (4) the formation of multiple charge states and/or adduct types during analysis. In theory, hydrolysates are more similar the lower the peptide length, especially when the protein source is similar and when the evolutionary divergence time (and rate) between source animals is low [15]. Typically, structural analysis of peptides in complex mixtures, such as digests or hydrolysates, is performed using non-targeted, data-dependent UPLC-MS/MS. In MS/MS, precursor ions are subjected to a fragmentation method, such as collision-induced dissociation or electron transfer dissociation [16, 17]. Structural analysis of short peptides is less straightforward than the analysis of longer peptides as the often singly charged short peptides require more energy to fragment [18], which frequently results in a decrease in sequence information through interresidue bond cleavages and in an increase of the formation of immonium ions [19]. Predicted fragmentations, e.g., for di- and tripeptides [20] and/or retention time prediction [21], are helpful to assign peptides. When MS data are extracted from a non-targeted hydrolysate data set, single m/z values will often generate multiple peaks in the chromatograms, which is especially true for collagens because their primary structure contains many slightly different repetitions. In many cases, permutations of the sequence will have to be considered as well as isomeric combinations of amino acids, which have also been explored by Wu and coworkers [22]. When the protein source of a hydrolysate is well characterized and relatively pure, many possible permutations can be dismissed. However, when the intended protein source is not pure, it might be necessary to consider all possibilities. The potential of incomplete sequence information in MS/MS then remains problematic, especially in relation to permutations and isomeric combinations. It is not possible, even with the aid of data analysis software, to always correctly assign the amino acid constituents and determine their order without confirmation using a reference.

This article will present the results of the non-targeted analysis of four different collagen hydrolysates in different matrices (solvent, TIM (TNO gastro-Intestinal Model) dialysate and human serum). We proceed by illustrating that a general overview of the data set can be obtained combining non-targeted and targeted data analysis. The insight gained by following such a top down (data) analytical workflow can be crucial for defining a suitable targeted analysis strategy.

## Materials and methods

**Scheme 1:**
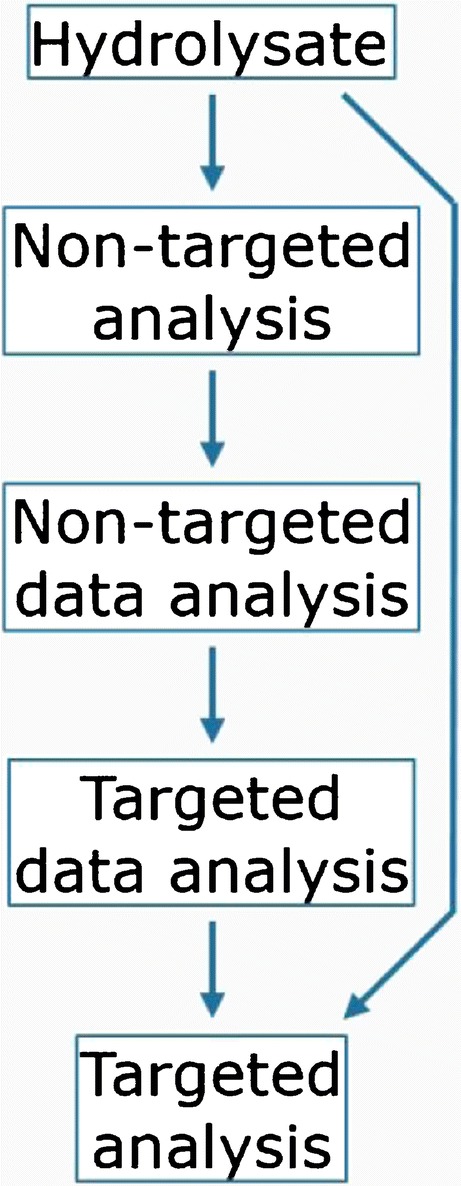
(data) Analytical flow scheme

Four products provided by Rousselot were tested (see Table 1). Collagen hydrolysates were analyzed using UPLC-MS. There were three types of samples, the dissolved start products (samples Px), TIM dialysates (samples Txy), and human serum (samples Sxy), to compare the state of the hydrolysates before and after absorption, i.e., uptake into the blood. Non-targeted analyses were performed with the P, T, and S samples and targeted analyses were performed with the S samples. The (data) analytical workflow is illustrated in Scheme 1. The second character *x* in sample codes denotes the product (A–D) and the third character *y* the spike status (S: sample (not spiked); L: spike low; and H: spike high).

**Table 1:** Information of the tested products

Product	Animal source	Source tissue	Average MW
A: Peptan B2000	Bovine	Hide	Low
B: Peptan P2000	Porcine	Skin	Low
C: Peptan P5000	Porcine	Skin	High
D: Peptan F2000	Fish species	Skin	Low

### TIM

Tiny-TIM [23] is a validated dynamic in vitro system that can be used for the determination of the true ileal (protein) digestibility and bioaccessibility. During the experiments, the digested and dissolved molecules are dialyzed from the intestinal lumen through a semipermeable membrane unit connected to the small intestinal compartment. This allows the assessment of the bioaccessible fraction in the dialysate, which is the fraction of nutrients and/or other compounds that are potentially available for small intestinal absorption. One product (A, B, C, or D) was added per tiny-TIM experiment using the conditions mentioned in Table 2. Average test conditions (healthy human adult) after the intake of a protein meal were simulated, such as gastric emptying [24]. Prior to each experiment, the secretion fluids (e.g., gastric juice with enzymes, electrolytes, bile, and pancreatic juice) were freshly prepared, the pH electrodes calibrated, and semipermeable membrane (hollow fiber) units installed. Pooled dialysate samples (0–360 min) were generated, snap-frozen, and stored at < − 70 °C. The total dialysate volume was approximately four liters.

**Table 2:** Overview of gastrointestinal parameters

Parameter	Measure
Stomach	
Intake (total)	150 g (water, artificial saliva, product and gastric start residue containing enzymes)
Intake (product)	8 g
Gastric half emptying time (t½)	50 min
Gastric pH	6.7 to 2.0 in 90 min
Enzymes	Amylase, lipase, pepsin
Small intestine	
Residence time	~ 360 min
Small intestinal pH	6.5
Secretions	Bile and pancreatic juice (containing trypsin, amylase, lipase, protease)
Temperature	37 °C ± 1 °C

### Serum samples

Twelve human volunteers ingested 25 g of products A–D (three volunteers per group). The serum collection was carried out following the rules of the Declaration of Helsinki. The human experiment was approved by the French Ethical Committee [Comité de Protection des Personnes (CPP17048/No. IDRCB: 2017-A02543-50) of Saint-Germain-en-Laye - Ile de France XI]. No negative effects were reported by collagen hydrolysate ingestion (one oral dose of 25 g diluted in 200 ml of water). The volunteers were informed of the objectives of the study and the potential risks of ingestion of collagen hydrolysate, such as diarrhea and abdominal pain. Non-targeted analysis was performed in serum samples drawn 60 min after ingestion. The dipeptides **p**G and P**p** and total hydroxyproline (denoted as Hyp or **p**) were quantified in serum samples drawn at *t* = 0 and 60 min after ingestion.

### Non-targeted analysis

P samples were prepared at 25 mg/ml in 100 mM ammonium bicarbonate (ABC, Fluka) in Milli-Q water (MQ, > 18 MΩ, Advantage A10). To 100 μl of the 25 mg/ml solutions, 50 μl 100 mM ABC was added. To 100 μl T sample, either 50 μl 100 mM ABC (sample) or 3.0 mg/ml (spike low) or 15 mg/ml (spike high) of the corresponding product (A, B, C, or D) in 100 mM ABC was added. To 100 μl S sample, either 50 μl 100 mM ABC (sample) or 1.5 mg/ml (spike low) or 15 mg/ml (spike high) of the corresponding product in 100 mM ABC was added. To all samples (with volume 150 μl), 450 μl of 1 μg/ml EVF (tripeptide injection standard) in methanol (Biosolve) containing 0.1% trifluoroacetic acid (Sigma-Aldrich) and 1% formic acid (FA, Biosolve) was added to perform a mild protein precipitation. After centrifugation for 5 min at 3500–14000 rpm, the supernatants were analyzed with high resolution UPLC-MS (Dionex Ultimate 3000 UHPLC – Thermo Q Exactive). The analytical column was an Acquity HSS T3, 100 × 2.1 mm, 1.8 μm (Waters), operated at 40 °C. The flow rate was 500 μl/min. Peptides were eluted from the analytical column using the following gradient: 0–1 min: 98% A (0.1% FA in MQ) and 2% B (0.1% FA in acetonitrile (ACN, Biosolve)); 1–12 min: 98–70% A and 2–30% B; 12–12.5 min: 70–5% A and 30–95% B; 12.5–13 min: 5% A and 95% B; 13–13.5 min: 5–98% A and 95–2% B; 13.5–18 min: 98% A and 2% B. The MS analyzer was operated in full scan positive mode, resolution 17,500. The injection volume was 3 μl.

### Targeted analysis

For the analysis of dipeptides **p**G and P**p**, 50 μl ice cold ACN was added to 50 μl serum while vortexing. After incubation for 10 min at room temperature, the samples were centrifuged at 14,000 rpm for 5 min, the supernatant diluted in borate buffer, derivatized with AccQ-Tag (Waters) [25], diluted with MQ, and analyzed using UPLC-MS (Acquity – Waters Xevo TQ-S). Matrix-matched calibration samples were prepared with **p**G and P**p** (Bachem) in pooled human serum (Bioreclamation) in a range of 0.04–40 μg/ml and prepared and analyzed using the same conditions. The analytical column was an Acquity HSS T3, 100 × 2.1 mm, 1.8 μm (Waters), operated at 60 °C. The flow rate was 600 μl/min. Peptides were eluted from the analytical column using the following gradient: 0–0.5 min: 97.5% A (0.1% FA in MQ) and 2.5% B (0.1% FA in ACN); 0.5–7.5 min: 97.5–60% A and 2.5–40% B; 7.5–8.0 min: 60–10% A and 40–90% B; 8.0–8.6 min: 10% A and 90% B; 8.6–8.7 min: 10–97.5% A and 90–2.5% B; 8.7–10 min: 97.5% A and 2.5% B. After positive electrospray ionization, m/z 359.14 > 189.10 (**p**G + AccQ-Tag [M + H]^+^ > y_2_^+^) was used for the quantification of **p**G and m/z 399.30 > 132.20 (P**p** + AccQ-Tag [M + H]^+^ > y_1_^+^) for P**p**. The injection volume was 5 μl. Total Hyp was determined by hydrolysis with hydrochloric acid (Merck) prior to derivatization with AccQ-Tag and UPLC-MS analysis. Matrix-matched calibration solutions were prepared using Hyp (Sigma-Aldrich) in a range of 0.2–200 μg/ml and prepared and analyzed using the same conditions. After positive electrospray ionization, m/z 302 > 171 (Hyp + AccQ-Tag [M + H]^+^ > AccQ-Tag^+^) was used for the quantification of Hyp.

## Results and discussion

The high numbers of hydrolysate components and their dynamics make it difficult to predict which peptide is interesting to analyze in relation to a research question and thus to predefine a strategy for a targeted analysis. Moreover, preconceptions about the possible outcome of a study can lead to a biased setup and unawareness of the actual coverage of the total potential outcome. Therefore, we have implemented the analytical strategy to first map data sets by performing non-targeted LC-MS analysis followed by non-targeted data analysis. Subsequently, targeted data analysis can reveal trends for individual (groups of) components and, finally, targeted analyses can be defined and performed to obtain quantitative data. Below, we present the results of this novel strategy to analyze hydrolysates in vitro and in vivo, applied to collagen hydrolysates, going from non-targeted towards targeted analysis, illustrating the analytical workflow and possible considerations.

### Non-targeted data analysis

**Fig. 1:**
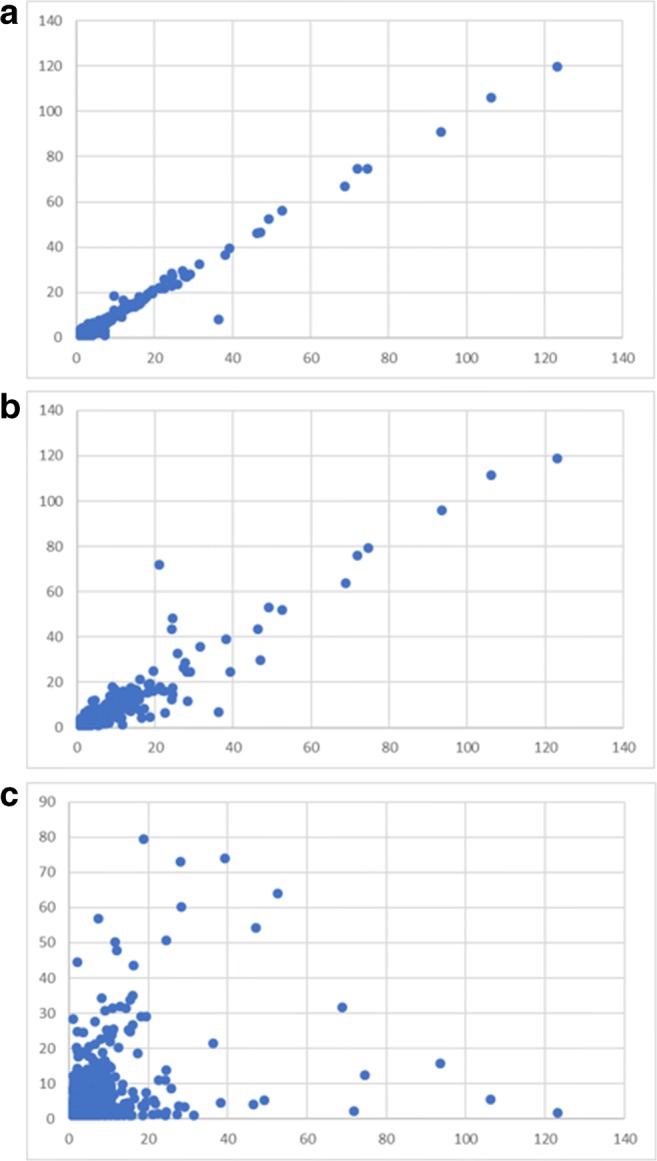
Signal ratios of collagen tryptic digests in the m/z 150–2000 range plotted per nominal m/z value. The x-axis sample is always mixed bovine/porcine limed bone collagen, plotted against **a** the same sample (replicate analysis), **b** mixed bovine/porcine (different ratio) limed bone collagen, and **c** pure bovine limed bone collagen sample

**Fig. 2:**
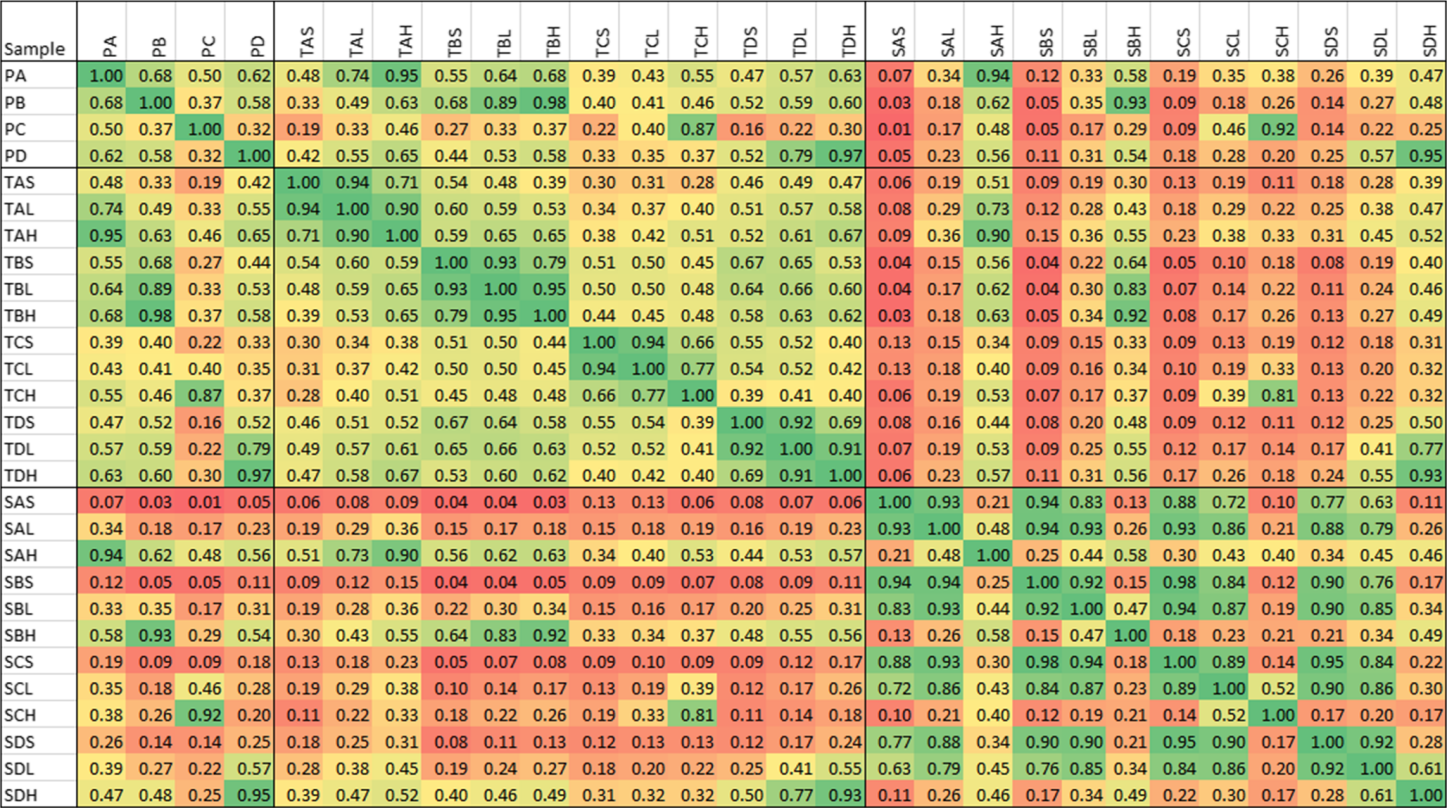
Correlation matrix between non-targeted data of pure products A–D, TIM dialysate samples, and serum samples. The sample codes are explained in the Materials and methods section

**Fig. 3:**
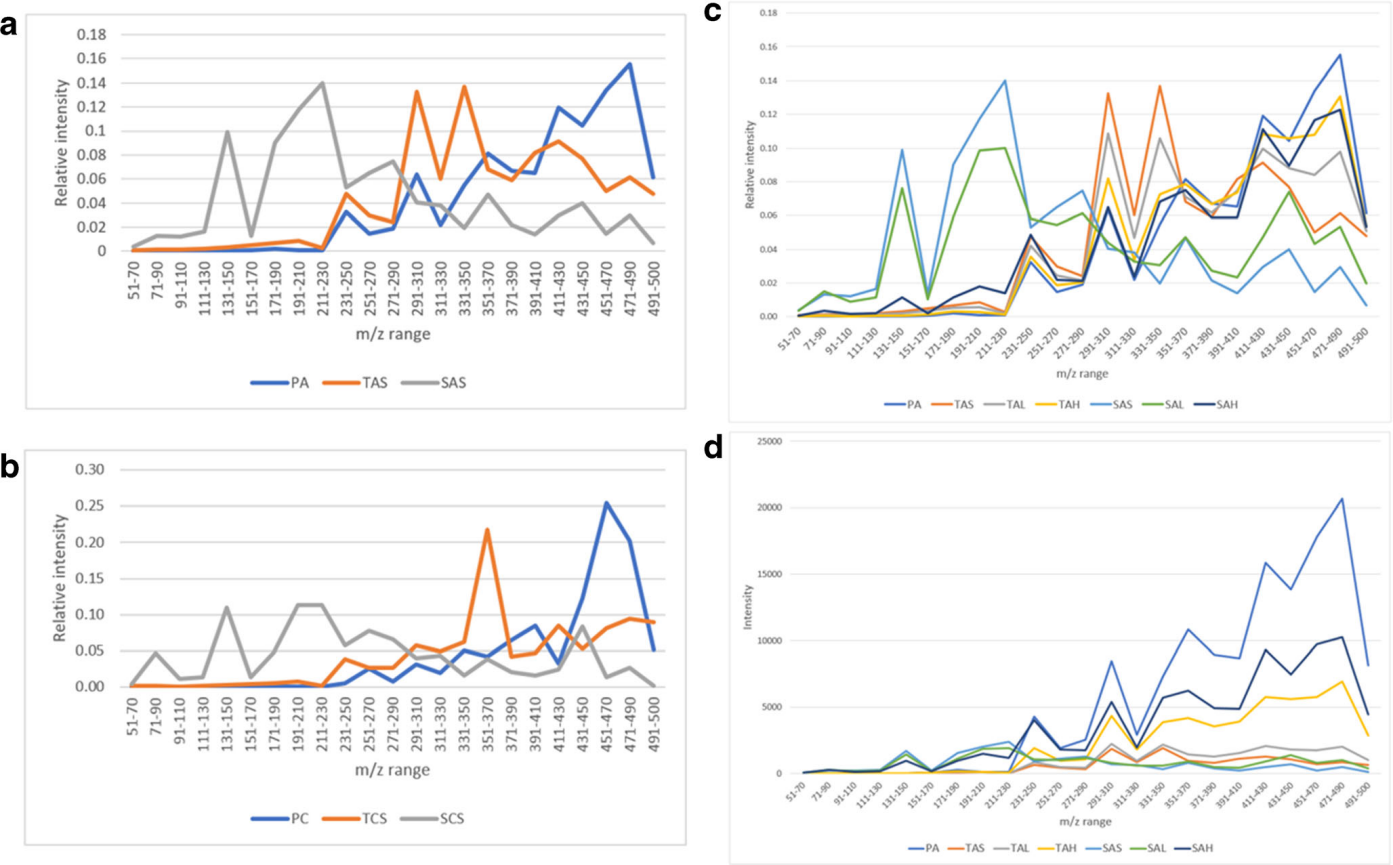
Relative signal ratios per m/z range for **a** product A (lower average MW), **b** product C (higher average MW), and **c** product A including spiked samples; **d** absolute signal ratios per m/z range of product A including spiked samples. M/z comparisons are not the same as molecular weight comparisons, but in general, the m/z value increases with increasing mass and below m/z 500 singly charged ions are abundant. The sample codes are explained in the Materials and methods section

Start products, TIM dialysates, and serum samples were analyzed by LC-MS as described in the Materials and methods section. Chromatograms were converted to MS spectra. MS data were exported as intensities per nominal m/z value. A solvent blank was used to correct for solvent background signals [26]. Division by the background signal provides smoother data than background signal subtraction. All signals related to solvent will give a value close to 1 and all signals related to the products will provide values > 1. The data used to correct for background signals is referred to as correction reference in the remaining part of the text and the outcome of the division of sample data by the correction reference data as signal ratios. An advantage of this approach is that a quick and global view of the similarities and differences between samples is obtained. In addition, the data have semi-quantitative properties (see Table 3). Especially in the cleaner matrices (product and TIM dialysate), the signal ratio sums show an overall good relation with the spiked concentration, except for the TCL sample. For serum, the relation with concentration seems to be less linear and this might be related to effects of the more complex matrix background and thus relatively lower product-related signals. We proceeded with assessing the similarity between the data of all the samples. To illustrate the nature of the sample comparisons, in Fig. 1, plots are presented of the signal ratios between a different set of samples (tryptic digests of collagen samples), showing an expected decreasing correlation with decreasing similarity. The correlation coefficients between the signal ratios per nominal m/z value, which do not depend on the product concentration, were calculated for each comparison and the individual coefficients were organized in a correlation matrix. In Fig. 2, the correlation coefficients between start product, TIM dialysate, and serum (average of 3 subjects per product) data are reported. All spikes and especially the high spikes correlate very well with the corresponding start product showing good analytical performance in each matrix. TIM dialysate samples did not have a high correlation with the corresponding start products which is expected due to the processing of components in the simulated gastrointestinal tract. This is especially true for the higher average molecular weight (MW) product C. TIM dialysate of product C exhibits more correlation with the shorter average MW products, which indicates that product components become more similar when the average MW becomes lower, as has been discussed in the Introduction and in the ESM. Although there is an underlying matrix background, in Fig. 3, the decrease in average MW is illustrated by the obtained signal ratios between m/z 51 and 500 divided in 23 smaller m/z ranges. Figure 3 clearly shows that the start products have a higher relative intensity at higher m/z values and serum samples at lower m/z values, while TIM dialysate samples are in between. The latter findings confirm that TIM is a suitable in vitro model to predict luminal gastrointestinal processing of ingested compounds in humans. From Fig. 2, it appears that the average MW has a more profound effect on similarity than the source animal (mainly for start product and TIM dialysate). Serum samples are similar to neither start product nor TIM dialysate samples, most probably due to further processing, selective uptake, and serum background. Because the same correction reference (solvent blank) was used to calculate all signal ratios, the comparison of serum samples with the other sample types is suboptimal. When samples in the same matrix are compared, it is possible to calculate signal ratios using a matrix-specific correction reference, such as blank TIM dialysate for TIM dialysate samples, to improve the comparison quality. For serum samples, which show high biological variation between individuals, in time and depending on circumstantial factors, it could be justified to use pooled serum or the minimum observed values in a set as correction reference.

**Table 3:** Signal ratio sum per sample type, related to theoretical end concentrations. The sample codes are explained in the Materials and methods section. The values in the right column were calculated using the signal ratio sum (3rd column), except for the spiked samples, where the corrected signal ratio sum (4th column) was used for calculation

Sample type	End concentration product (mg/ml) (relative to T spike high)	Signal ratio sum	Signal ratio sum corrected for sample background	Relative to corrected T spike high
PA	4.17 [3.33]	133,082	–	3.39
PB		149,661	–	3.65
PC		92,765	–	4.29
PD		149,094	–	3.65
TAS	–	14,021	–	0.36
TBS		11,448	–	0.28
TCS		13,796	–	0.64
TDS		13,620	–	0.33
TAL	0.25 [0.20]	20,631	6609	0.17
TBL		18,848	7400	0.18
TCL		32,877	19,081	0.88
TDL		21,087	7467	0.18
TAH	1.25 [1.00]	53,279	39,258	1.00
TBH		52,420	40,971	1.00
TCH		35,405	21,608	1.00
TDH		54,478	40,858	1.00
SAS	–	17,252	–	0.44
SBS		15,259	–	0.37
SCS		14,105	–	0.65
SDS		12,864	–	0.31
SAL	0.125 [0.10]	18,981	1729	0.04
SBL		19,336	4077	0.10
SCL		15,810	1705	0.08
SDL		16,950	4086	0.10
SAH	1.25 [1.00]	83,552	66,300	1.69
SBH		103,461	88,201	2.15
SCH		58,294	44,189	2.05
SDH		99,153	86,289	2.11

### Targeted data analysis

Quantitative LC-MS measurements and unambiguous structure confirmation often require either suitable internal standardization, external standardization, or standard addition for each analyte of interest. When targeted data analysis is performed on a non-targeted data set, the quantification issue will arise again, because the data are then considered on an individual component basis. An intermediate form of targeted data analysis can be to consider component groups. To be able to relate signals to start product components and express them as product concentration equivalent, samples spiked with product can be analyzed. Spikes at different concentration levels can be used to assess matrix effect and linearity in the spiked concentration range, on an individual component basis, but also on component group basis. Using this approach, it can be investigated whether components are more abundant before or after processing in a semi-quantitative fashion.

In the data set, we observed components which provided signal in the start product and in the spiked samples but not in the TIM dialysate. These components are most probably not bioaccessible or they are degraded in the TIM system. In addition, there are components which provided a signal in the TIM dialysate but not in the start product. The latter components are most probably formed by digestion in the TIM system and they are bioaccessible. As an example, nominal m/z 357 is considered (see Table 4). The dominant signal in this channel originates from m/z 357.213, which represents the tetrapeptide AGJP (and isomers, where J stands for (iso)leucine). In theory A, P, and J could be removed from AGJP during digestion to form collagen tripeptides. Therefore, nominal m/z 286, 260, and 244, dominated by respective signals at 286.176, 260.161, and 244.129, were also considered. The four products exhibit different component landscapes and it should be noted that m/z 357 is also an intermediate which can be formed from larger peptides. It is assumed that for each start product, the component landscapes merely shift through the presently recorded state and will become more similar regarding MW distributions when they end up in the blood, as illustrated by Fig. 3.

**Table 4:** Signal ratios of the four products (PA–PD), TIM blank dialysate (T bl), non-spiked dialysate sample (TxS), dialysate samples spiked at 3.0 mg/ml (TxL, end concentration 0.25 mg/ml), dialysate samples spiked at 15 mg/ml (TxH, end concentration 1.25 mg/ml), dialysate sample signal ratios corrected for blank dialysate (Corr stands for corrected), the outcome of the division of product signal ratios by TxS Corr (12.5 is the end concentration ratio when 100% would go to the dialysate; values < 12.5 indicate that the TIM dialysate contains more than product equivalent; values > 12.5 indicate that the TIM dialysate contains less than product equivalent). Finally, two quality parameters, which can be used to assess whether an obtained result is analytically acceptable: the additional signals for spike low and spike high samples and the outcome of the division (value close to 5 indicates linear signal in the concentration range) and the product signal ratios divided by the additional signals for spike high (should be 3.33 when there is no matrix effect)

Sample	m/z 244	m/z 260	m/z 286	m/z 357	Sum/division of sums
PA	783.88	534.29	34.31	708.25	2060.73
PB	33.29	2243.26	85.06	699.29	3060.90
PC	82.50	116.99	47.06	128.09	374.65
PD	21.90	4903.73	282.45	995.51	6203.59
T bl	1.46	25.57	4.59	0.94	32.56
TAS	15.11	108.68	26.95	80.74	231.49
TAL	41.52	133.09	26.03	118.56	319.19
TAH	189.84	258.89	59.30	287.85	795.88
TAS Corr (TAS–T bl)	13.64	83.11	22.36	79.81	198.92
TAL Corr (TAL–T bl)	40.05	107.52	21.44	117.62	286.63
TAH Corr (TAH–T bl)	188.38	233.31	54.71	286.91	763.32
PA/TAS Corr	57.46	6.43	1.53	8.87	*10.36*
**A** TAL Corr–TAS Corr	26.41	24.41	− 0.93	37.81	87.70
**B** TAH Corr–TAS Corr	174.73	150.20	32.35	207.10	564.39
**B**/**A**	6.62	6.15	− 34.92	5.48	*6.44*
PA/**B**	4.49	3.56	1.06	3.42	*3.65*
TBS	5.59	219.72	11.48	37.60	274.38
TBL	7.65	338.42	15.41	68.69	430.17
TBH	12.76	912.95	42.41	217.31	1185.44
TBS Corr (TBS–T bl)	4.12	194.15	6.89	36.67	241.82
TBL Corr (TBL–T bl)	6.19	312.84	10.82	67.76	397.61
TBH Corr (TBH–T bl)	11.30	887.38	37.82	216.38	1152.87
PB/TBS Corr	8.07	11.55	12.35	19.07	*12.66*
**A** TBL Corr–TBS Corr	2.07	118.70	3.93	31.09	155.79
**B** TBH Corr–TBS Corr	7.18	693.23	30.93	179.71	911.05
**B**/**A**	3.47	5.84	7.86	5.78	*5.85*
PB/**B**	4.64	3.24	2.75	3.89	*3.36*
TCS	6.26	39.07	25.21	31.08	101.61
TCL	9.03	92.93	57.44	86.43	245.82
TCH	29.49	60.75	38.96	58.56	187.75
TCS Corr (TCS–T bl)	4.80	13.50	20.62	30.14	69.05
TCL Corr (TCL–T bl)	7.57	67.35	52.85	85.49	213.26
TCH Corr (TCH–T bl)	28.02	35.17	34.37	57.62	155.18
PC/TCS Corr	17.20	8.67	2.28	4.25	*5.43*
**A** TCL Corr–TCS Corr	2.77	53.86	32.23	55.35	144.21
**B** TCH Corr–TCS Corr	23.23	21.68	13.75	27.48	86.13
**B**/**A**	8.39	0.40	0.43	0.50	*0.60*
PC/**B**	3.55	5.40	3.42	4.66	*4.35*
TDS	13.94	476.60	23.77	89.13	603.43
TDL	10.53	792.01	41.80	133.70	978.04
TDH	20.55	1945.92	131.05	323.37	2420.88
TDS Corr (TDS–T bl)	12.48	451.02	19.18	88.19	570.87
TDL Corr (TDL–T bl)	9.07	766.43	37.21	132.76	945.48
TDH Corr (TDH–T bl)	19.09	1920.34	126.46	322.43	2388.32
PD/TDS Corr	1.76	10.87	14.73	11.29	*10.87*
**A** TDL Corr–TDS Corr	− 3.41	315.41	18.03	44.57	374.61
**B** TDH Corr–TDS Corr	6.61	1469.32	107.28	234.24	1817.45
**B**/**A**	− 1.94	4.66	5.95	5.26	*4.85*
PD/**B**	3.31	3.34	2.63	4.25	*3.41*

For collagen hydrolysates, peptides containing hydroxyproline are of special interest as hydroxyproline is a characteristic amino acid in collagens and because Hyp-containing di- and tripeptides have been reported to carry specific bioactivity that might relate to health benefits of collagen peptide supplementation. The non-targeted data gave the impression that short peptides containing hydroxyproline provided either a low signal or that hydroxyproline might be predominantly present in longer peptides, because many theoretically abundant short peptides containing hydroxyproline, such as **p**, **p**G, A**p**, P**p**, GA**p**, GP**p**, A**p**G, P**p**G, **p**GA, **p**GE, and **p**GP, were not observed with high intensity in the non-targeted data. The latter hypothesis (Hyp in longer peptides) is less probable, because we assumed that through the action of proteolytic enzymes and due to the enzymatic and chemical processing which takes place in the TIM system, also short peptides containing hydroxyproline should be formed. Therefore, we decided to perform a targeted analysis. A solution to enhance signals of hydroxyproline(-containing short peptides) is to derivatizate with AccQ-Tag. This reagent binds to amine groups of amino acids and short peptides, is easily protonated, and enhances the chromatographic properties.

### Targeted analysis

Total Hyp (after hydrolysis) and the theoretically most abundant Hyp-containing dipeptides from collagen, P**p,** and **p**G were analyzed in a targeted fashion in the serum samples. For Hyp, a bioanalytically acceptable calibration curve was obtained, meaning that the back-calculated analyte concentrations of 75% of the calibration samples were within ± 15% (± 20% at the lowest calibration level) of the theoretical value, in the 1–200 μg/ml range after correction for the level found in the pooled human serum. It is important to note that at *t* = 60 min the mean increase in total Hyp concentration was approximately 14.5 μg/ml (110 nmol/ml). The dipeptides **p**G and P**p** which could be carriers of Hyp to the blood were determined at *t* = 0 and *t* = 60 min. The mean **p**G concentration increased from less than 0.04 μg/ml to 0.66 μg/ml after 1 h (increase 3.5 nmol/ml) and the mean P**p** concentration increased from 0.23 to 2.3 μg/ml after 1 h (increase 9.1 nmol/ml). It can be concluded that, in this experimental setup, **p**G and especially P**p** contribute significantly as carrier to the total Hyp increase in blood after ingestion of collagen hydrolysate. The values determined for total Hyp, **p**G, and P**p** in this study fit with values reported earlier in literature [27–30]. The previously reported values, however, vary to a great extent, regarding the ratio between free Hyp and peptide-bound Hyp and the relative concentrations of different Hyp carriers. After ingestion of collagen hydrolysate, Yazaki et al. [27] mainly found the Hyp carriers GP**p** in murine plasma and mainly P**p** in skin. Similarly, Iwai et al. [28] mainly found P**p** and lesser amounts of A**p**, A**p**G, P**p**G, L**p**, I**p**, and F**p** in human serum and plasma. The major component that Ichikawa et al. [29] found in human plasma was P**p** and minor components were A**p**G, S**p**G, A**p**, F**p**, L**p**, I**p**, GP**p**, and P**p**G. Finally, Taga et al. [30] particularly found increased X**p**G (where X represents any amino acid) in murine plasma when they administered a gelatin hydrolysate that was produced using a cysteine-type ginger protease. Differences in the findings of (previous) studies can be the result of (minor) differences in the experimental set up. In the present study, a complete and unique picture was obtained by LC-MS analysis of the dissolved start products, in vitro-generated dialysates (containing the digested components that are potentially available for small intestinal absorption), and human serum collected after product ingestion. We have shown that the four tested collagen hydrolysates exhibit different component landscapes during the course of digestion and absorption. There are many possible Hyp carriers: several tripeptides and other dipeptides [29]. The abundance of particular carriers will depend on the extent of hydrolysis of the start product and the subsequent in vivo processing, e.g., the chemical and enzymatic processing which takes place in the gastric and intestinal compartments, as shown by our in vitro-generated TIM data. Finally, also brush border enzyme activity and selective transfer to the blood compartment will play a role. Targeted analysis can be very helpful to investigate the concentration of Hyp carriers. When there is a limited number of analytes to be determined, acceptable standardization can be achieved through synthesis of proper (internal) standards at acceptable costs.

## Conclusion

In the present study, four collagen hydrolysates were analyzed in different matrices (solvent, TIM dialysate, and human serum) using non-targeted LC-MS, to compare the state of the hydrolysates before and after absorption, either simulated in vitro or in vivo. The presented (data) analytical workflow is especially useful for investigating the behavior of protein hydrolysates. Prior to ingestion, their composition is often already highly complex, and during the course of digestion and absorption, hydrolysate components will be exposed to several enzymes (with broad specificity) and to chemical hydrolysis. Many hydrolysate components will be formed from precursors and be degraded at the same time, which decreases the utility of recording concentrations of intermediates. We illustrated that a general overview of the data set can be obtained by combining non-targeted and targeted data analysis. The insight gained by following such a top down (data) analytical workflow can be crucial for defining a suitable targeted setup and considering data trends beyond the defined targets.

## Electronic supplementary material

ESM 1 (PDF 216 kb)
