# Supplementary material for: Non-targeted and targeted analysis of collagen hydrolysates during the course of digestion and absorption
Source: Anal Bioanal Chem. 2019 Dec 24;412(4):973–82. doi: 10.1007/s00216-019-02323-x (PMC7005076; doi:10.1007/s00216-019-02323-x)
Supplement: Supplementary file 1 — (PDF 216 kb) [file 216_2019_2323_MOESM1_ESM.pdf]

Analytical and Bioanalytical Chemistry

Electronic Supplementary Material

Non-targeted and targeted analysis of collagen hydrolysates during the course of digestion and absorption

Anne J. Kleinnijenhuis, Frédérique L. van Holthoon, Annet J.H. Maathuis,
Barbara Vanhoecke, Janne Prawitt, Fabien Wauquier, Yohann Wittrant

Supplementary Information

A typical mass spectrum of a collagen hydrolysate (see Fig. S1) exhibits a multitude of molecular species. The maximum number of peptides per peptide length *p* from a linear protein sequence with length *n* is (*n*-(*p*-1)). The number of detected molecular species increases when 1) partial modification sites are present, either modified *in vivo* or by the sample preparation, 2) multiple charge states and/or adduct types are formed during analysis and 3) originally there were non-linear structure elements present. Theoretically, hydrolysates are more similar the lower the peptide length, especially when the protein source is similar and when the evolutionary divergence time (and rate) between source animals is low [1]. Assuming that a protein source contains the 20 most common amino acids, hydrolysates become at maximum 20 times more similar when *p* decreases by one, either by processing prior to ingestion or by the chemical and enzymatic reactions that take place *in vivo*. In many cases it is still possible to discriminate between protein sources after extensive hydrolysis using the relative abundances of hydrolysate components.

It is a challenging task to perform comprehensive structural analysis of hydrolysate peptides. Typically, structural analysis of peptides in complex mixtures, such as digests or hydrolysates, is performed using non-targeted ultra-performance liquid chromatography – mass spectrometry (UPLC-MS) and data-dependent MS/MS. In MS/MS, precursor ions are subjected to a fragmentation method, such as collision induced dissociation or electron transfer dissociation [2, 3]. After structural identification, ideally a reference is used to match precursor ion m/z, retention time and MS/MS fragmentation for ultimate confirmation, but this is not feasible when there are multiple analytes of interest, due to the synthesis costs. It is relatively straightforward to identify longer, often multiply charged tryptic peptides based on MS/MS data. The shorter peptides present in hydrolysates are often singly charged. The energy required to fragment ions increases with decreasing charge [4]. Application of high collision energies results in more complex fragmentation and, often, a decrease in sequence information through interresidue bond cleavages. Fortunately, at higher collision energies another type of peptide fragment ion is formed with higher intensity, the so-called immonium ions [5], which are amino acid specific internal fragment ions. Immonium ions are very useful for (partial) determination of the amino acid content of short peptides. In addition y_1 and a_2 in combination with b_2 ions can provide useful information as these peptide fragments contain the C- and N- termini. Finally, predicted fragmentations, e.g. for di- and tripeptides [6] and/or retention time prediction [7] are helpful to assign peptides.

When MS data are extracted from a non-targeted hydrolysate data set, single m/z values will often generate multiple peaks in the chromatograms, which is especially true for collagens because their primary structure contains many slightly different repetitions. Amino acid constituents within a peptide with a given mass can occur in different sequences, e.g. a tripeptide containing A, G and R might occur as GAR, GRA, ARG, AGR, RAG and RGA. In fact, all these six combinations occur in bovine collagen 1α1 and 1α2, the two proteins that are present in the collagen type 1 triple helix, see Table S1. In principle there are $n!$ permutations for a peptide containing n different, known amino acid constituents. When r known amino acid constituents within a sequence are the same, there are $n!/r!$ permutations [8]. Presence of an isomeric isoleucine or leucine residue will double the number of permutations. A complicating factor is that a number of amino acid combinations have exactly the same mass. In Table S2 the isomeric combinations of amino acids and dipeptides, relevant for collagens, have been summarized, which has also been partly explored by Wu and coworkers [9]. In Fig. S2 the occurrence of GI / GL / IG / LG / AV / VA in the sequence of bovine collagen 1α1 is illustrated and Fig. S3 shows an extracted chromatogram of the corresponding m/z 189.12 in a collagen hydrolysate, illustrating that multiple species are detected. When the protein source of a hydrolysate is well characterized and relatively pure, many possible permutations can be dismissed. However, when the intended protein source is not pure, it might be necessary to consider all possibilities. The potential of incomplete sequence information in MS/MS then remains problematic, especially in relation to permutations and isomeric combinations. It is not possible, even with the aid of data analysis software, to always correctly assign the amino acid constituents and determine their order without confirmation using a reference.

References

[1] Kleinnijenhuis AJ. Visualization of Genetic Drift Processes Using the Conserved Collagen 1α1 GXY Domain. J Mol Evol. 2019;87:106-130.

[2] Syka JE, Coon JJ, Schroeder MJ, Shabanowitz J, Hunt DF. Peptide and protein sequence analysis by electron transfer dissociation mass spectrometry. Proc Natl Acad Sci USA. 2004;101:9528–9533.

[3] Kleinnijenhuis AJ, Hedegaard C, Lundvig D, Sundbye S, Issinger OG, Jensen ON, Jensen PH. Identification of multiple post-translational modifications in the porcine brain specific p25alpha. J Neurochem. 2008;106:925-933.

[4] Loo JA, Edmonds CG, Smith RD. Tandem mass spectrometry of very large molecules: serum albumin sequence information from multiply charged ions formed by electrospray ionization. Anal Chem. 1991;63:2488-2499.

[5] Olsen JV, Macek B, Lange O, Makarov A, Horning S, Mann M. Higher-energy C-trap dissociation for peptide modification analysis. Nat Methods. 2007;4:709-712.

[6] Tang Y, Li R, Lin G, Li L. PEP Search in MyCompoundID: Detection and Identification of Dipeptides and Tripeptides Using Dimethyl Labeling and Hydrophilic Interaction Liquid Chromatography Tandem Mass Spectrometry. Anal Chem. 2014;86:3568-3574.

[7] Moruz L, Käll L. Peptide retention time prediction. Mass Spec Rev. 2017;36:615–623.

[8] Pólya G. Kombinatorische Anzahlbestimmungen für Gruppen, Graphen und chemische Verbindungen. Acta Mathematica 1937;68:145–254.

[9] Wu M, Xu Y, Fitch WL, Zheng M, Merritt RE, Shrager JB, Zhang W, Dill DL, Peltz G, Hoang CD. Liquid Chromatography/Mass Spectrometry Methods for Measuring Dipeptide Abundance in Non-Small Cell Lung Cancer. Rapid Commun Mass Spectrom. 2013;27:2091–2098.

Table S1 Abundance of AGR permutations (m/z 303.178) in bovine collagen 1α1 and 1α2

Tripeptide	Number of occurrences in bovine collagen 1α1	Number of occurrences in bovine collagen 1α2
GAR	9	10
GRA	0	1
ARG	9	10
AGR	3	2
RAG	0	1
RGA	5	4

Table S2 isomeric amino acid combinations, only considering amino acids and dipeptides. J stands for (iso)leucine, **k** for hydroxylysine and **p** for hydroxyproline

Combi 1	Combi 2	Combi 3	Monoisotopic mass $[M+H]^+$ ions
I	L	-	132.102
GG	N	-	133.061
AG	Q	-	147.077
AS	GT	-	177.088
AV	GJ	-	189.124
A**p**	PS	-	203.103
AN	GQ	-	204.098
AD	EG	-	205.082
AE	**p**S	-	219.098
JS	TV	-	219.134
AM	CV	-	221.096
DV	**p**T	-	233.114
NT	QS	-	234.109
A**k**	KS	-	234.145
DT	ES	-	235.093
EP	**pp**	-	245.114
JN	QV	-	246.145
DJ	EV	-	247.129
AY	FS	-	253.119
JQ	K**p**	**k**P	260.161
DQ	EN	-	262.104
EK	**kp**	-	276.156
F**p**	PY	-	279.134
EF	**p**Y	-	295.129
F**k**	KY	-	310.177
HY	NW	-	319.141

a)

b)

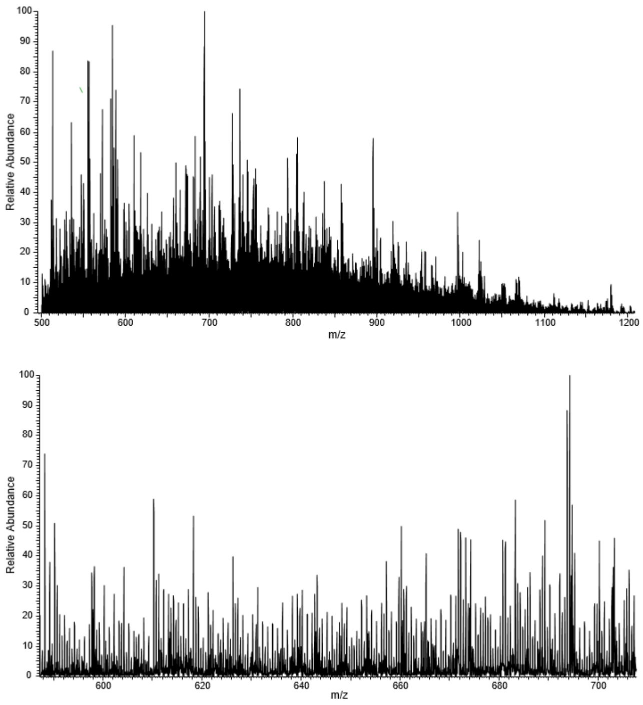

Fig. S1 MS spectrum of a) product D in the 2-9 minute range and b) zoom m/z 600-700

```
MFSFVDLRLLLLLAATALLTHGQEEGQEEGQEEDIPPVTCVQNGLRYHDRDVWKPVPCQI
CVCDNGNVLCDDVICDELKDCPNAKVPTDECCPVCPEGQESPTDQETTGVEGPKGDTGPR
GPRGPAGPPGRDGIPGQPGLPGPPGPPGPPGPPGLGGNFAPQLSYGYDEKSTGISVPGPM
GPSGPRGLPGPPGAPGPQGFQGPPGEPGEPGASGPMGPRGPPGPPGKNGDDGEAGKPGRP
GERGPPGPQGARGLPGTAGLPGMKGHRGFSGLDGAKGDAGPAGPKGEPGSPGENGAPGQM  300
GPRGLPGERGRPGAPGPAGARGNDGATGAAGPPGPTGPAGPPGFPGAVGAKGEGGPQGPR
GSEGPQGVRGEPGPPGPAGAAGPAGNPGADGQPGAKGANGAPGIAGAPGFPGARGPSGPQ
GPSGPPGPKGNSGEPGAPGSKGDTGAKGEPGPTGIQGPPGPAGEEGKRGARGEPGPAGLP
GPPGERGGPGSRGFPGADGVAGPKGPAGERGAPGPAGPKGSPGEAGRPGEAGLPGAKGLT
GSPGSPGPDGKTGPPGPAGQDGRPGPPGPPGARGQAGVMGFPGPKGAAGEPGKAGERGVP  600
GPPGAVGPAGKDGEAGAQGPPGPAGPAGERGEQGPAGSPGFQGLPGPAGPPGEAGKPGEQ
GVPGDLGAPGPSGARGERGFPGERGVQGPPGPAGPRGANGAPGNDGAKGDAGAPGAPGSQ
GAPGLQGMPGERGAAGLPGPKGDRGDAGPKGADGAPGKDGVRGLTGPIGPPGPAGAPGDK
GEAGPSGPAGPTGARGAPGDRGEPGPPGPAGFAGPPGADGQPGAKGEPGDAGAKGDAGPP
GPAGPAGPPGPIGNVGAPGPKGARGSAGPPGATGFPGAAGRVGPPGPSGNAGPPGPPGPA  900
GKEGSKGPRGETGPAGRPGEVGPPGPPGPAGEKGAPGADGPAGAPGTPGPQGIAGQRGVV
GLPGQRGERGFPGLPGPSGEPGKQGPSGASGERGPPGPMGPPGLAGPPGESGREGAPGAE
GSPGRDGSPGAKGDRGETGPAGPPGAPGAPGAPGPVGPAGKSGDRGETGPAGPAGPIGPV
GARGPAGPQGPRGDKGETGEQGDRGIKGHRGFSGLQGPPGPPGSPGEQGPSGASGPAGPR
GPPGSAGSPGKDGLNGLPGPIGPPGPRGRTGDAGPAGPPGPPGPPGPPGPPSGGYDLSFL 1200
PQPPQEKAHDGGRYYRADDANVVRDRDLEVDTTLKSLSQQIENIRSPEGSRKNPARTCRD
LKMCHSDWKSGEYWIDPNQGCNLDAIKVFCNMETGETCVYPTQPSVAQKNWYISKNPKEK
RHVWYGESMTGGFQFEYGGQGSDPADVAIQLTFLRLMSTEASQNITYHCKNSVAYMDQQT
GNLKKALLLQGSNEIEIRAEGNSRFTYSVTYDGCTSHTGAWGKTVIEYKTTKTSRLPIID
VAPLDVGAPDQEFGFDVGPACFL
```

Fig. S2 occurrence of GI / GL / IG / LG / AV / VA (m/z 189.124) in the bovine collagen 1α1 sequence

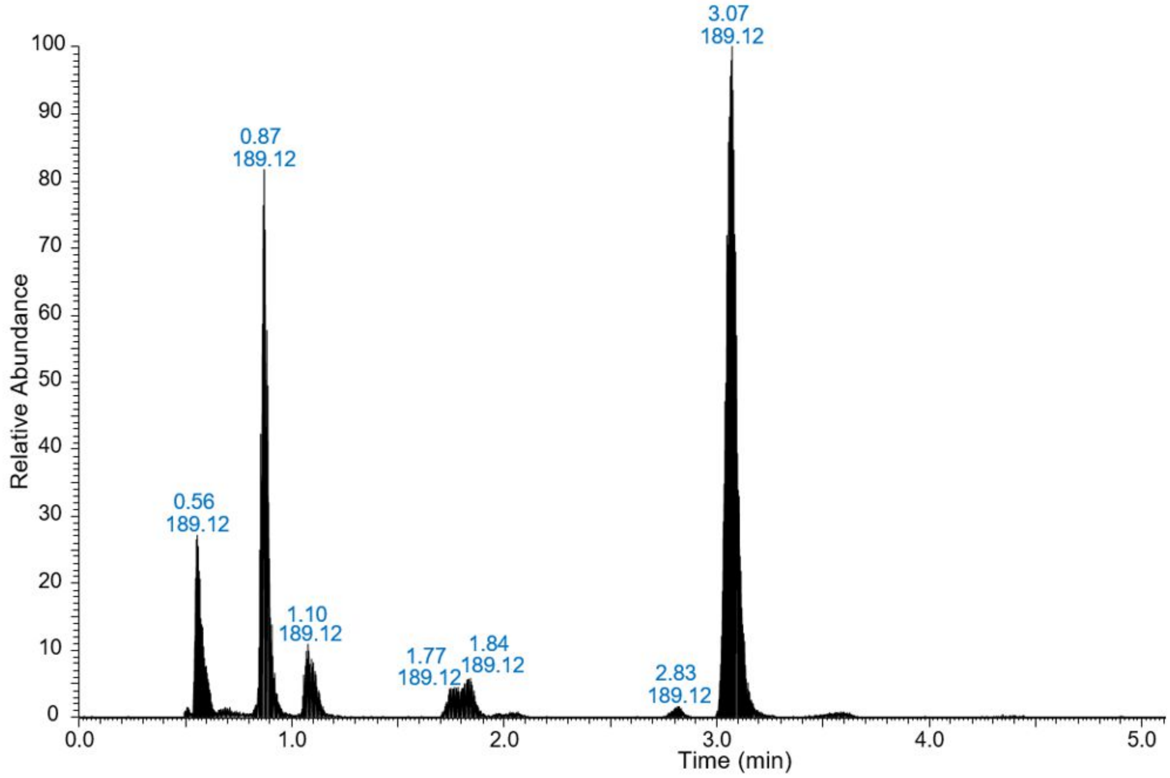

Fig. S3 extracted chromatogram of m/z 189.12 in product D
